# Complete Genome Sequence of a Non-Carbapenemase-Producing Carbapenem-Resistant Providencia rettgeri Strain Isolated from a Clinical Urine Sample in Arkansas

**DOI:** 10.1128/mra.00474-22

**Published:** 2022-07-27

**Authors:** Zulema Udaondo, Kaleb Z. Abram, Trent Gulley, Kelley Garner, Jennifer Shray, Mary Whisnant, Ashley Harris-Spotts, Maui Crawford, Atul Kothari, Thidathip Wongsurawat, Sun-Hee Moon, En Huang, Se-Ran Jun

**Affiliations:** a Department of Biomedical Informatics, University of Arkansas for Medical Sciences, Little Rock, Arkansas, USA; b Healthcare-Associated Infection Program, Arkansas Department of Health, Little Rock, Arkansas, USA; c Arkansas Public Health Laboratory, Arkansas Department of Health, Little Rock, Arkansas, USA; d Department of Environmental and Occupational Health, University of Arkansas for Medical Sciences, Little Rock, Arkansas, USA; University of Maryland School of Medicine

## Abstract

Here, we report the complete genome sequence of Providencia rettgeri isolate PROV_UAMS_01, which was recovered in 2021 from a urine sample from a hospitalized patient in Arkansas, USA. The genome sequence of P. rettgeri isolate PROV_UAMS_01 comprises a single chromosomal replicon with a G+C content of 40.51% and a total of 3,887 genes.

## ANNOUNCEMENT

Here, we present the complete genome sequence of a Providencia rettgeri clinical isolate with a carbapenem-resistant antibiogram that is also resistant to ampicillin, aztreonam, cefazolin, nitrofurantoin, piperacillin and tazobactam, and tetracycline. The isolate was collected from a urine sample from a hospitalized patient in Faulkner County, AK. Culturing of the urine sample on a blood agar plate yielded *P. rettgeri*, which was confirmed by matrix-assisted laser desorption ionization–time of flight (MALDI-TOF) mass spectrometry (Bruker Biotyper; Bruker Daltonics, USA). Antimicrobial susceptibility testing was performed using the Vitek 2 system (bioMérieux) with the AST-GN card. Detection of carbapenemase production was negative based on the modified carbapenem inactivation method (mCIM) (1), following the CLSI guidelines (2). Culture samples of this strain were submitted to the Arkansas Department of Health directly. Therefore, there was no direct contact with the study participant. The Institutional Review Board (IRB) classified this study as exempt (IRB No. 261022).

Genomic DNA was extracted, purified, and sequenced as described in references 3 and 4. Briefly, genomic DNA was extracted using pure colonies of *P. rettgeri* subcultured for 24 h on blood agar plates. The colonies were resuspended into a DNA/RNA Shield collection and lysis tube. Then, genomic DNA was extracted using the Quick-DNA fungal/bacterial kit (Zymo Research, Irvine, CA, USA) and further purified using AMPure XP beads (Beckman Coulter). The DNA concentration was quantified and quality controlled using a NanoDrop spectrophotometer, the Agilent 2200 TapeStation system, and a Qubit 3.0 fluorometer (Thermo Fisher Scientific). The purified DNA was aliquoted into two tubes for MinION and Illumina sequencing.

An Oxford Nanopore Technologies (ONT) sequencing library was prepared using a PCR-free method of multiplexing samples with the rapid barcoding kit (SQK-RAD004); the library was sequenced using a FLO-MIN106 (R9.4) flow cell for 48 h. The short-read sequencing library was sequenced using the DNBSEQ-G400 platform at BGI Genomics (San Jose, CA), where they followed their standard protocol to construct DNA libraries of 2 × 150-bp paired-end reads. Reads with adapter contamination and low-quality reads with a base quality score of <Q20 were filtered out using SOAPnuke software (5).

Adapter sequences were trimmed from the short paired-end reads using fastp v0.23.2 (6). The quality of the pre- and postprocessed reads was assessed using the FastQC tool v0.11.9 (7). Long-read base calling and demultiplexing were conducted using the model dna_r9.4.1_450bps in Guppy v4.5.4 (8), with min_qscore set to 9. Adapters were trimmed from the Nanopore long reads using Porechop v0.2.4 (https://github.com/rrwick/Porechop). Nanopore read quality control was performed using NanoFilt v2.3 and NanoStat v1.5.0 from NanoPack (9). A *de novo* hybrid assembly was built using Unicycler v0.4.8 (10), with the ONT long reads and paired-end short reads as the input, which resulted in one circular chromosome without any identified plasmids. The absence of plasmid replicons was further confirmed using the KMA algorithm in PlasmidFinder v2.0.1 against the *Enterobacterales* plasmid database v2021-11-29 (11). Default parameters were used for all software unless otherwise specified.

The complete circular chromosome was rotated with Unicycler, using the *dnaA* gene as the starting gene, and annotated using the NCBI Prokaryotic Genome Annotation Pipeline (PGAP) v5.3 (12). A BLAST alignment of the seven complete 16S rRNA genes (1,528 bp) identified in isolate PROV_UAMS_01 against the NCBI nonredundant/nucleotide (nr/nt) database showed 99.93 to 100% sequence similarity with the 16S rRNA genes from other *P. rettgeri* strains. Assignment of PROV_UAMS_01 to the species *P. rettgeri* was further confirmed as in reference 13 using Mash v2.3 (14) to calculate the genomic distance of isolate PROV_UAMS_01 against a local database of all *Providencia* genomes available in GenBank (220 genomes as of 13 March 2021). A summary of the whole-genome sequencing data and the main genomic features of isolate PROV_UAMS_01 are listed in Table 1.

**TABLE 1:** Sequencing summary of Providencia rettgeri isolate PROV_UAMS_01

Characteristic	Data
Genome	
Yr of isolation	2021
Source	Arkansas Department of Health
Illumina sequencing	
No. of reads	3,521,034
Size (bp)	528,155,100
Avg coverage (×)	150
SRA accession no.	SRR17269190
ONT sequencing	
No. of reads	598,721.0
Size (bp)	2,144,262,328.0
Read N50 (bp)	5,805.0
Median read length (bp)	2,464.0
Avg coverage (×)	503
SRA accession no.	SRR17269189
Assembly	
Assembler	Unicycler v0.4.8
No. of scaffolds	1
Total genome size (bp)	4,266,731
Chromosome size (bp)	4,266,731
G+C content (%)	40.51
Total no. of genes	3,887
Total no. of CDSs^*a*^	3,783
No. of genes (coding)	3,754
No. of CDSs (with protein)	3,754
No. of genes (RNA)	104
No. of rRNAs (5S, 16S, 23S)	8, 7, 7
No. of complete rRNAs (5S, 16S, 23S)	8, 7, 7
No. of tRNAs	78
No. of ncRNAs^*b*^	4
Total no. of pseudogenes	29
No. of confirmed CRISPRs	0
No. of plasmids	0
GenBank accession no.	CP090005.1
BioSample accession no.	SAMN24180573
BioProject accession no.	PRJNA789997

a CDSs, coding sequences.

b ncRNAs, non-coding RNAs.

### Data availability.

The complete genome assembly of *P. rettgeri* isolate PROV_UAMS_01 was deposited in DDBJ/ENA/GenBank under the accession number CP090005.1. The long and short reads are available in the NCBI SRA database under the accession numbers SRR17269189 and SRR17269190, respectively.
